# Synergistic Effects of MTHFR, MTRR, and MTR Gene Variants on Serum Folate Levels and Cognitive Function in Chinese Preschoolers: A Cross-Sectional Study

**DOI:** 10.3390/nu17162666

**Published:** 2025-08-18

**Authors:** Lingling Ou, Luolan Peng, Jingbo Wang, Chao Han, Xiayu Zhao, Mengyao Wang, Mengtian Wang, Zhaolong Gong, Yan Li

**Affiliations:** 1 National Institute for Nutrition and Health, Chinese Center for Disease Control and Prevention, Beijing 100050, China; ling000uoo@163.com (L.O.); wangjb@ninh.chinacdc.cn (J.W.); hanchao@ninh.chinacdc.cn (C.H.); zhaoxy@ninh.chinacdc.cn (X.Z.); romona123@163.com (M.W.); wangmengtian2001@163.com (M.W.); 2 People’s Medical Publishing House Research Institute, Beijing 100021, China; pengll0504@163.com; 3 Fengtai District Center for Disease Control and Prevention of Beijing Municipality, Beijing 100071, China

**Keywords:** MTHFR, MTR, MTRR, folate, cognition, preschool children

## Abstract

**Background/Objectives**: Subnormal folate levels have a detrimental impact on the growth and development of preschoolers. We aimed to investigate the association between independent/synergistic effects of the gene polymorphisms (methyltetrahydrofolate reductase (MTHFR) *C677T* and *A1298C* polymorphisms, alongside methionine synthase reductase (MTRR) *A66G* polymorphism and the methionine synthase (MTR) *A2756G* polymorphism) and serum folate levels as well as cognitive levels in Chinese preschoolers aged 5–7 years. **Methods**: Data were sourced from 614 children, acquired through the “Long-term Health Effects Assessment Project of Infants and Toddlers Nutritional Pack (LHEAITNP)” program were used. Folate serum concentrations were measured using a microbiologically modified technique. The genotypes of MTHFR *A1298C* and *C677T*, together with MTRR *A66G*, were identified by Kramer’s Allele-Specific PCR (KASP) technique. The cognitive scores of children were assessed by questionnaire. **Results**: MTHFR *677TT* and MTR *2756AG + GG* correlated negatively with serum folate levels (*TT* vs. *CC + CT*, *p* = 0.0009 and *AG + GG* vs. *AA*, *p* = 0.0057, respectively). MTHFR *C677T* and *A1298C* were independently linked to an elevated risk of suboptimal cognitive development (*TT* vs. *CC + CT*, *p* = 0.0009 and *AA* vs. *CA + CC*, *p* < 0.0001, respectively). The joint impact of these risk genotypes showed significantly increased risk of folate deficiency and inferior cognitive function compared to non-risk genotypes, particularly in those with more than two risk genotypes. The findings were corroborated by a cumulative effects model (*p* < 0.05). **Conclusions**: Our results indicate the substantial association between folate-homocysteine metabolism gene variants and serum folate status/cognitive performance in Chinese preschoolers. Potential gene-nutrient interactions worthy of longitudinal investigation.

## 1. Introduction

Nutrition, particularly vitamins, significantly influences neurological and physiological development from early infancy through later life [1]. Folate (Vitamin B9) is a vital water-soluble B vitamin that is a transporter of one-carbon units in the body [2]. The metabolic pathway critically regulates DNA and protein synthesis, both vital processes for cellular proliferation and differentiation [3]. Impairments in these fundamental processes may lead to growth retardation and developmental anomalies [4]. The folate cycle is an intricate biochemical mechanism strongly linked to homocysteine (Hcy) metabolism [5,6,7]. Folate affects nerve myelination [8,9], whereas Hcy accumulation resulting from folate deficiency can cause structural damage to white matter [10], both of which affect cognitive performance [11]. Longitudinal genomic analyses reveal that DNA methylation profiles undergo substantial remodeling during the initial five years of life, achieving stability by age seven [12]. DNA methylation significantly correlates with nutrients, mainly when methyl production is modulated by specific nutrients within the one-carbon metabolic pathway (folate, methionine, and B12) [13,14].

Folate is assimilated in the small intestine and transformed into dihydrofolate (DHF) and tetrahydrofolate (THF). THF is further converted to 5,10-methylenetetrahydrofolate (5,10-CH_2_-THF), which is then turned into 5-methyltetrahydrofolate (5-MTHF) by the enzyme 5,10-methylenetetrahydrofolate reductase (MTHFR) [15,16,17,18]. 5-MTHF functions as a methyl donor, facilitating the transfer of a methyl group to homocysteine via the enzyme 5-methyltetrahydrofolate transmethylase (MTR) to produce methionine [19,20]. The activity of MTR relies on reducing 5-methyltetrahydrofolate transmethylase reductase (MTRR) to enable the cycle’s progression [21].

**Figure 1:**
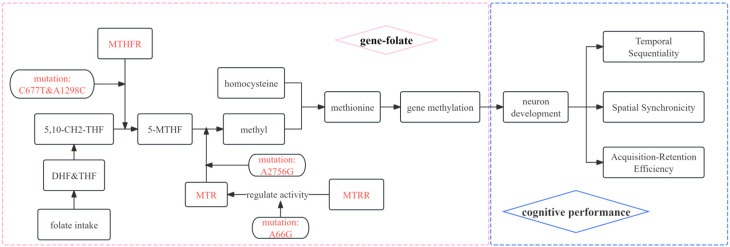
Association mechanism between gene–folate metabolic pathway and cognitive function.

Given this fundamental role of folate metabolism in cellular processes (Figure 1), we hypothesized that high-risk alleles for the single-nucleotide polymorphisms (SNPs) we studied (MTHFR *C677T*, MTHFR *A1298C*, MTRR *A66G*, and MTR *A2756G*) were correlated with suboptimal folate status/cognitive performance. However, research has examined the association between serum folate levels/cognitive performance and genetic variants, most focused on adult populations, with few studies on large samples of typical pediatric populations [22]. The impact of these polymorphisms throughout critical cognition-developmental periods (preschool age) remains unknown. To address this knowledge gap, we conducted a cross-sectional study to investigate the associations between the polymorphisms of these genes and serum folate levels/neurocognitive development.

## 2. Materials and Methods

### 2.1. Study Design

The population-based cross-sectional study was conducted in the provinces of Guizhou and Yunnan. All measurements (cognitive assessments and covariates) were acquired during a single study visit in 2023.

### 2.2. Research Participants

**Figure 2:**
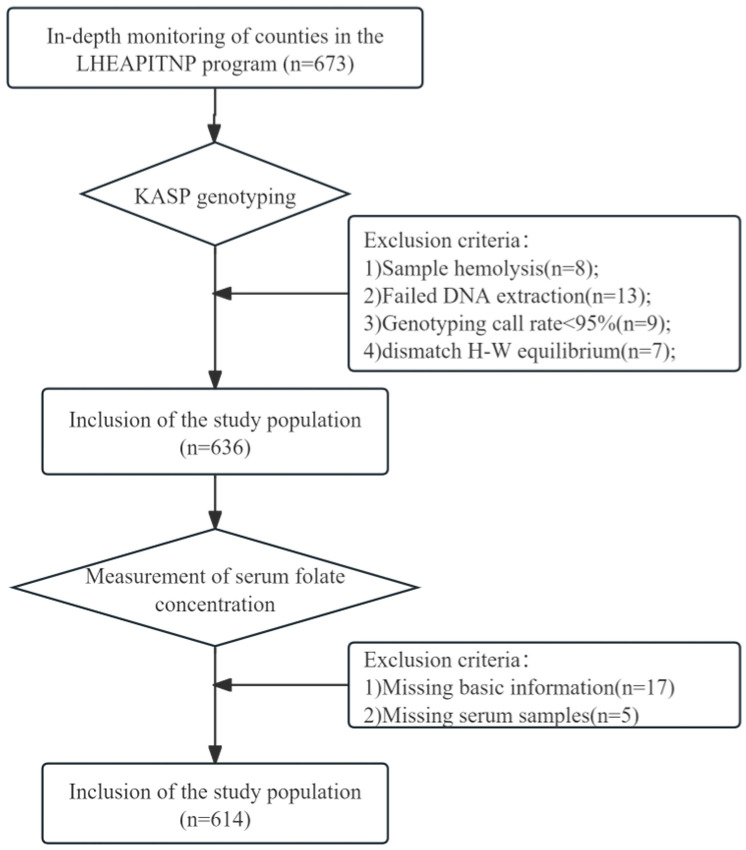
Inclusion and exclusion of study participants.

The study focused on the “Long-term Health Effects Assessment Project of Infants and Toddlers Nutritional Pack (LHEAITNP)” project. The participants were Han Chinese preschool children aged 5–7 years, all from the in-depth monitoring counties (Guiding County, Guizhou Province, and Song County, Henan Province). Figure 2 shows the detailed inclusion and exclusion criteria. A questionnaire was employed to gather fundamental information. The LHEAITNP project received approval from the Ethics Committee of the Institute of Nutrition and Health, Chinese Center for Disease Control and Prevention (No. 2018.017). All children’s guardians provided written informed consent before answering the questionnaire and collecting the biological specimens.

### 2.3. Blood Sample Dispensing and Storage

Researchers collected venous blood from participants in non-fasting states using vacuum blood collection tubes, maintained the samples at room temperature for 20–30 min, and centrifuged them at 3000 r/min for 15 min to separate the serum. The supernatant was aliquoted into a freezing tube and stored at −80 °C, while the sedimented blood cells were retained in the blood collection tube and stored at −20 °C.

### 2.4. DNA Extraction and Genotyping

DNA was extracted from frozen blood cell pellets using magnetic beads as the solid phase for nucleic acid adsorption. The Kramer’s allele-specific PCR (KASP) method was used for genotyping. We designed the KASP primers (Table 1) for four SNPs (MTHFR *C677T*/*A1298C*, MTRR *A66G*, and MTR *A2756G*, respectively) [23].

### 2.5. Measurement of Serum Folate Concentration

The improved microbial assay determined serum folate by measuring the growth reaction of Lactobacillus rhamnosus [24]. The process involves (1) preparing folate casein medium (for growth/detection purposes); (2) activating and preserving the bacterial strain; (3) inoculating the activated bacteria onto the detection medium and incubating the mixture with samples in a 96-well plate at 37 °C for 42–45 h; and (4) measuring the absorbance at 590 nm and calculating the concentration using the standard curve. The main form of serum folate detected by this method is 5-MTHF, which can accurately reflect the folate nutritional status of the human body.

### 2.6. Evaluation of Basic Cognition

According to “Wechsler Intelligence Scale for Children (WISC)”, three sections: “Graphical Reasoning”, “Reciting Numbers in Reverse Order”, and “Decoding”, are designed to assess children’s basic cognitive ability. The three components yielded a cumulative score of 100 for the three components (Supplementary Guidance S1).

### 2.7. Statistical Analysis

All statistical analyses were conducted by SAS (v. 9.4) statistical software, and data visualization was performed using R 4.5.1. Based on the non-normal distribution of continuous variables (age, BMI, birth weight/height), they are presented as median and quartiles, the differences of which were assessed using the Kruskal–Wallis test. Categorical variables (gender, province, caregiver’s education level) are presented as the number of instances (*n*) and percentages (%), and their prevalences were compared using the χ^2^ test. Low folate levels are defined as serum folate levels < 6 ng/mL, and high folate levels are defined as >20 ng/mL [25]. Groups based on serum folate level thresholds enable comparison of baseline characteristics across different folate status categories. Serum folate levels and cognitive scores show a skewed distribution, so further analysis was conducted after logarithmic transformation. Hardy–Weinberg equilibrium analysis of different genes was also performed through the χ^2^ test.

A linear regression model assessed the relationship between the independent and combined effects of gene polymorphisms and logarithmically transformed variables. Based on the comparison of baseline characteristics, we used age, gender, and the variables (BMI, birth weight, and province), which were different among groups, as covariates in adjusting the models. A generalized linear model (GLM) was used for trend testing to validate the above results. In the results, a two-sided *p* < 0.05 is considered statistically significant.

## 3. Results

### 3.1. Hardy–Weinberg Equilibrium Test

Six hundred and fourteen preschool children (boys: 315, girls: 299) were included, and their anthropometric data were collected. The four polymorphisms (MTHFR *C677T*, MTHFR *A1298C*, MTRR *A66G*, and MTR *A2756G*) are all consistent with the Hardy–Weinberg equilibrium (*p*-values of 0.1635, 0.2339, 0.8924, and 0.0855, respectively), indicating that the study participants derived from a homogeneous Mendelian population.

### 3.2. Demographic and Clinical Characteristics

No substantial differences were observed in age, birth height, cognitive scores, gender, and educational level of caregivers across the three levels of serum folate. However, children with higher BMI or birth weight exhibited reduced serum folate levels (*p* < 0.05). Statistically significant disparities exist in serum folate levels among children from different provinces (*p* < 0.0001). Comparison of genotype frequencies for four SNPs across varying folate levels revealed statistically significant differences for MTHFR *C677T* (*p* < 0.0001) and MTHFR *A1298C* (*p* = 0.0234) among the three groups (Table 2).

### 3.3. Association Between Genotypes and Variables (Folate, Cognitive Scores)

#### 3.3.1. Single Gene Polymorphism

Table 3 illustrates the association between MTHFR, MTRR, MTR polymorphisms, and serum folate levels, along with cognitive scores. For MTHFR *C677T*, the *TT* genotype was strongly associated with lower folate levels and cognitive scores compared to *CC* in analyses (adjusted β_folate_ = −0.0907, *p* = 0.0018; β_scores_ = −0.1253, *p* = 0.0002). *TT* genotypes also showed lower folate levels and cognitive scores than combining *CC + CT* genotypes (adjusted β_folate_ = −0.1595, *p* = 0.0009; β_scores_ = −0.0914, *p* = 0.0009). Children with MTHFR *1298AA*/*CA* had lower cognitive scores than those with either MTHFR *1298CC* genotype (Adjusted β_folate_ = −0.1165, *p* < 0.0001). No significant associations were found for MTRR *A66G*, while children carrying MTR *2756AG/GG* genotypes had lower serum folate levels than *AA* (Adjusted β_folate_ = −0.1402, *p* = 0.0057).

**Figure 3:**
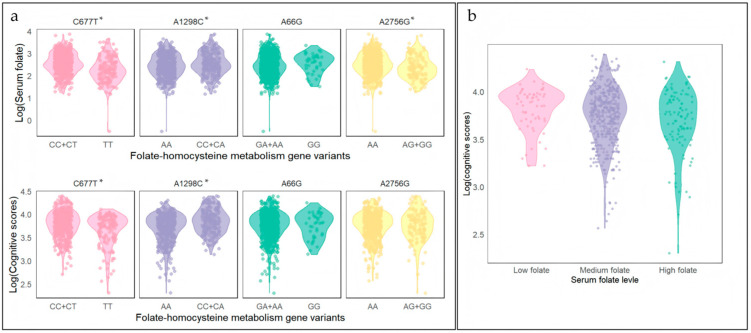
Association analysis between gene variants, serum folate levels, and cognitive scores. (**a**) single gene and folate/cognition; (**b**) folate and cognition. * *p* < 0.05.

Figure 3 illustrates the differences in serum folate/cognitive levels between genotypes and the differences in cognitive levels across serum folate subgroups. The serum folate levels and cognitive scores in individuals with the MTHFR *677TT*/*1298AA* genotype were significantly lower (*p* < 0.05). Serum folate levels were lower in subjects with the MTR *2756GG + AG* genotypes than those with MTR *2756AA* (*p* < 0.05). No significant differences in cognitive performance were observed across the MTRR *A66G* and MTR *A2756G* genotype variants. Cognitive scores remained comparable across the spectrum of serum folate levels.

#### 3.3.2. Joint Gene Polymorphisms

In joint analyses (Table 4), MTHFR *677TT*/*1298AA* genotype carriers exhibited reduced serum folate levels and cognitive scores compared to those with MTHFR *677CC + CT/1298CC + CA* (adjusted: β_folate_ = −0.1788, *p* = 0.0013; β_scores_ = −0.1538, *p* < 0.0001). Furthermore, children with MTHFR *677TT*/MTRR *GA + AA* genotypes had reduced folate levels and cognitive scores, in comparison to those with MTHFR *677CC + CT*/MTRR *66GG* genotypes (adjusted: β_folate_ = −0.2264, *p* = 0.0231; β_scores_ = −0.1169, *p* = 0.0401). Similarly, compared with the MTHFR *677CC + CT*/MTR *2756AA* carriers, children with MTHFR *677TT*/MTR *2756AG + GG* genotypes exhibited diminished serum folate levels and cognitive scores (Adjusted: β_folate_ = −0.2812, *p* = 0.0020; β_scores_ = −0.1253, *p* = 0.0165), as did MTHFR *1298AA*/MTR *2756AG + GG* carriers compared to *1298CC + CA*/MTR *2756AA* (adjusted: β_folate_ = −0.2172, *p* = 0.0017; β_scores_ = −0.1144, *p* = 0.0035). No substantial interactions were identified between MTRR *A66G* and MTHFR *A1298C*/MTR *A2756G*.

**Figure 4:**
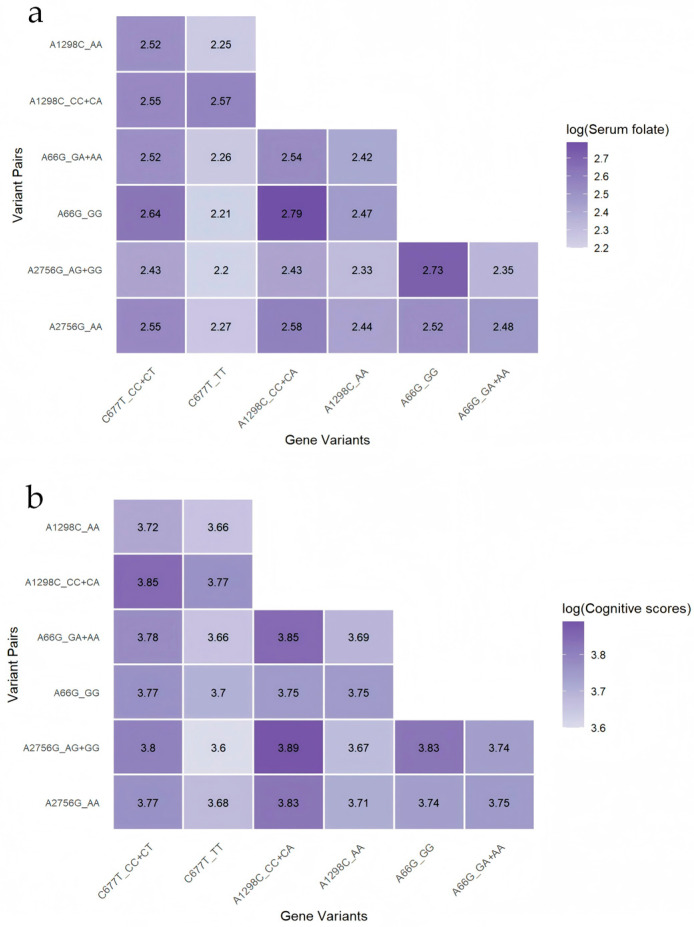
Logarithmic serum folate levels/cognitive scores by combined genotypes. (**a**) combined genotypes and folate; (**b**) combined genotypes and cognition.

Figure 4 demonstrates the relationship between pairwise gene combinations and serum folate levels/cognitive scores, revealing that combinations of two risk genotypes are associated with lower folate levels/cognitive performance.

### 3.4. Cumulative Genotypes and Serum Folate and Cognitive Scores

The correlation between cumulative risk genotypes and serum folate level/cognitive scores is shown in Table 5. In comparison to null risk genotype carriers, children possessing two (β = −0.1504, *p* < 0.05) or more risk genotypes (β = −0.2617, *p* < 0.05) had significantly lower serum folate levels. Additionally, the trend test results indicated a significant decrease in serum folate levels with an increasing quantity of risk genotypes (*p* < 0.05). Moreover, children’s basic cognitive scores declined as the quantity of risk genes increased (*p* < 0.05). Stratified analysis of folate levels indicated that children in the high folate level subgroup carrying two (β = −0.2231, *p* < 0.05) or more (β = −0.3644, *p* < 0.05) risk genotypes had reduced cognitive levels.

## 4. Discussion

Many studies have been conducted on folate metabolism-related SNPs. This study assessed the serum folate concentrations, basic cognitive scores, and genotypic frequencies of four SNPs in children to evaluate potential risk or interaction.

“Folates” is a broad category encompassing folic acid and its biologically active derivatives [2]. Aberrant folate metabolism is linked to various diseases [26]. A prevalent cause of megaloblastic anemia is folate insufficiency [27], which also leads to neural tube defects, cardiovascular diseases, and cognitive impairment [28,29]. Early childhood development constitutes a phase of exceptional neuroplasticity wherein cognitive, socioemotional, communicative, and motor systems undergo coordinated maturation throughout the brain’s primary growth period (0–8 years) [30]. Folate is strongly correlated with the neural cognitive development of children [31,32], so the polymorphisms of folate metabolism-related genes are relevant [33].

MTHFR, a pivotal folate metabolic enzyme, regulates circulating folate homeostasis. Its functional polymorphisms (*C677T* and *A1298C*) directly impair enzymatic activity, disrupting folate metabolism [34,35]. The *T* allele of the MTHFR *C677T* variant would reduce MTHFR activity due to increased enzyme thermolability [36]. MTHFR-mediated 5-MTHF production from 5,10-CH2-THF is compromised by the MTHFR *C677T* variant [37], leading to impaired homocysteine metabolism and consequent hyperhomocysteinemia [18]. Elevated homocysteine accelerates folate depletion. This study indicated that compared with MTHFR *677CC* genotype carriers, children with MTHFR *677TT* genotype exhibited reduced serum folate levels (*p* = 0.0018, Table 3) and diminished cognitive scores (*p* = 0.0002, Table 3), corroborated by many studies [38,39,40,41]. Furthermore, our findings showed that the synergistic effect of MTHFR *677TT* and *1298AA* genotype correlated with decreased cognitive scores (*p* < 0.0001, Table 4) and serum folate levels (*p* = 0.0013, Table 4), corroborating findings by Zappacosta B et al. [34], suggesting that the interaction of the two MTHFR polymorphisms significantly reflected serum folate status and may consequently impact cognitive development in children.

The *A1298C* variant (*C* allele) causes an alanine–glutamate substitution in MTHFR, disrupting the conversion of methyl tetrahydrofolate to tetrahydrobiopterin and subsequently affecting folate concentrations [42]. This study revealed a significant disparity in cognitive scores associated with different genotypes of MTHFR *A1298C*. Children with the MTHFR *1298AA* genotype exhibited lower serum folate levels than those with the MTHFR *1298CC + CA* genotype (*p* = 0.0866, Table 3). Also, they demonstrated lower cognitive scores (*p* < 0.0001, Table 3), indicating that the MTHFR *1298CC + CA* genotype may be a protective factor against folate deficiency. In conjunction with studies that have obtained the same results, it is indicated that the C allele of the MTHFR *1298* polymorphism correlates with an increased risk of elevated folate concentrations, thus decreasing the risk of cognitive disorders [43,44].

MTR and MTRR are pivotal enzymes in the folate-homocysteine metabolism pathway, regulating folate cycling and methylation processes through synergistic activity [45]. A deficiency in MTRR leads to the inactivation of MTR, preventing the conversion of 5-MTHF to THF, ultimately disrupting the folate metabolic cycle and influencing nerve cell differentiation, synapse formation, and myelination [46]. In the study, the MTRR *A66G* polymorphism shows no significant association with serum folate levels/cognitive scores in children across *GG*, *GA*, and *AA* genotypes (*p* > 0.05, Table 3), a finding corroborated by Nasri K et al. [15]. Potential explanations for the non-significant association include a healthy dietary state obscuring the effect and inadequate statistical power of the study (limited sample size of mutant genotype *GG*), among others.

MTR dysfunction inhibits 5-MTHF conversion to THF, disrupting folate cycling and causing functional deficiency [47]. This study showed that children with MTR *2756AG + GG* had a lower serum folate level than the others (*p* = 0.0057, Table 3). Conversely, research by Li W et al. [48] and Barbosa PR et al. [49] has demonstrated an increased risk of folate deficiency compared to the MTR *2756GG* genotype. The joint effects of MTR *A2756G* and MTRR *A66G* polymorphisms exhibited a non-significant association with serum folate levels or cognitive performance. However, the combinations of MTHFR *C677T* and MTRR *A66G* (*p* < 0.05, Table 4), as well as MTHFR *C677T*/*A1298C* and MTR *A2756G* (*p* < 0.05, Table 4), exhibited significant correlations, which implied disrupted methyl flux: reduced 5-MTHF production (MTHFR defect) coupled with impaired THF regeneration (MTR defect) depleted serum folate pools [50].

The study identified the risk genotypes (MTHFR *677TT*, MTHFR *1298AA*, MTRR *66GA+AA*, and MTR *2756AG + GG*, respectively) associated with lower serum folate levels and cognitive scores. However, this study’s cognitive scores under the high folate level stratum were lower overall than those under the low folate level stratum (Figure 3b). The potential explanation for the paradoxical finding may be: (1) the potential “methyl trap” phenomenon at elevated folate concentrations may disrupt vitamin B12 metabolism and exacerbate cognitive impairment [51]; (2) serum folate levels do not accurately reflect 5-MTHF levels in neural tissue [52]. Future investigations should incorporate erythrocyte folate, B12 status, and methylation biomarkers to characterize this gene-nutrient interaction better.

Our assessment revealed that as the severity of genetic defects increases, so does the risk of serum folate deficiency/poor cognitive performance in children (*p* < 0.05). Subgroup analysis revealed that the higher folate level group results were consistent with the above findings (*p* < 0.05, Table 4). The potential cause could be that serum folate reflects recent intake, thus failing to assess actual tissue utilization appropriately.

The primary novelty of this study is its concentration on Chinese preschoolers as the study population, highlighting early-life genetic vulnerability as a key factor of serum folate variation during periods of rapid growth. However, our study has several important limitations: (1) the regional focus of the st on two provinces may limit generalizability, warranting future larger regional scale and joint economic status investigation to validate; (2) the lack of vitamin B12 assessment represents a key limitation given its metabolic interplay with folate. Future work should integrate B12 measurement, homocysteine analysis, and B12-related genetic variants for more robust evaluation.

## 5. Conclusions

Folate insufficiency has been consistently linked to cognitive dysfunction. Our study confirmed that the MTHFR *677TT* homozygous variant was statistically associated with folate deficiency/poor cognitive performance. Notably, we observed that each combination was associated with folate levels and the cognitive function through a comprehensive analysis of polymorphic interactions between (i) MTHFR *C677T* and *A1298C*, (ii) the MTHFR *C677T* and MTRR *A66G*, (iii) the MTHFR *C677T* and MTR *A2756G*, and (iv) MTHFR *A1298C* and MTR *A2756G*. Future studies could explore the potential value of genetic screening in folate monitoring and systematically assess its translational feasibility.

## Figures and Tables

**Table 1:** KASP primers for four SNPs.

SNP	Primer FAM	Primer HEX	Universal Primers
MTHFR *C677T*	*GCTCCGTCATGATGAAATCGG*	*GCTGCGTCATCATCAAATCGA*	*CTGACCTGAACCACTTGAAGGA*
MTHFR *A1298C*	*GGAGGAGCTGACCAGTCAAGA*	*GGAGGAGCTGACCAGTGAAGC*	*GGTAAAGAACGAAGACTTCAAAGACACTT*
MTRR *A66G*	*GGCAAAGGCCATCGCAGAAGAAATA*	*GGCAAAGGCCATCGCAGAAGAAATG*	*TGAAGATCTGCAGAAAATCCATGTACC*
MTR *A2756G*	*CCTTGAGAGACTCATAATGGC*	*CCTTGAGAGACTCATAATGGT*	*CTTTGAGGAAATCATCGAAGAA*

**Table 2:** Sociodemographic and epidemiologic characteristics of the preschool children.

Variables			Serum Folate Level ^a^		*p*-Value ^b^
		Low (n = 59)	Medium (n = 453)	High (n = 102)	
		M (q1–q3) or n (%)	M (q1–q3) or n (%)	M (q1–q3) or n (%)	
Age (months)		72 (68–77)	72 (68–77)	72 (68–77)	0.4279
BMI		14.9 (14.2–16.5)	14.5 (13.7–15.6)	14.4 (13.5–15.3)	0.0051
Birth weight (g)		3350 (3030–3800)	3245 (3000–3550)	3200 (3000–3400)	0.0496
Birth height (cm)		50 (50–51)	50 (50–50)	50 (50–50)	0.1447
Cognitive scores		49 (40–53)	44 (35–52)	43 (34–52)	0.0805
serum folate level (ng/mL)		5.1 (4.4–5.4)	11.3 (8.6–14.3)	23.6 (21.8–26.6)	<0.0001
Gender	Male	37 (62.71)	233 (51.43)	45 (44.12)	0.0748
	Female	22 (37.29)	220 (48.57)	57 (55.88)	
Province	Henan Province	45 (76.27)	246 (54.30)	27 (26.47)	<0.0001
	Guizhou Province	14 (23.73)	207 (45.70)	75 (73.53)	
Educational level of caregivers	Primary school or below	16 (27.12)	134 (29.58)	33 (32.35)	0.9690
	Junior high school	32 (54.24)	238 (52.54)	51 (50.00)	
	High school or above	11 (18.64)	81 (17.88)	18 (17.65)	
MTHFR *C677T*	*CC*	6 (10.17)	127 (28.04)	37 (36.27)	<0.0001
	*CT*	25 (42.37)	215 (47.46)	52 (50.98)	
	*TT*	28 (47.46)	111 (24.50)	13 (12.75)	
MTHFR *A1298C*	*CC*	1 (1.69)	20 (4.42)	6 (5.88)	0.0234
	*CA*	11 (18.64)	126 (27.81)	40 (39.22)	
	*AA*	47 (79.66)	307 (67.77)	56 (54.90)	
MTRR *A66G*	*GG*	3 (5.08)	31 (6.84)	5 (4.90)	0.3982
	*GA*	26 (44.07)	162 (35.76)	46 (45.10)	
	*AA*	30 (50.85)	260 (57.40)	51 (50.00)	
MTR *A2756G*	*AA*	47 (79.66)	370 (81.68)	84 (82.35)	0.3706
	*AG*	10 (16.95)	78 (17.22)	15 (14.71)	
	*GG*	2 (3.39)	5 (1.10)	3 (2.94)	

^a^ Serum folate levels subgroup criteria: low: <6 ng/mL; medium: 6–20 ng/mL; high: >20 ng/mL; ^b^ Kruskal–Wallis or Pearson χ^2^ tests were used to examine differences in continuous variables and in proportions, respectively, between serum folate status groups.

**Table 3:** Associations of gene polymorphisms with serum folate level/cognitive scores.

Variables		n (%)	Adjusted β (95%CI) ^a,b^	Adjusted β (95%CI) ^a,b^
MTHFR *C677T*	*CC*	170 (27.69)	reference	reference
	*CT*	292 (47.56)	−0.0318 (−0.1250, 0.0615)	−0.0491 (−0.1023, 0.0041) +
	*TT*	152 (24.76)	−0.0907 (−0.1476, −0.0338) *	−0.1253 (−0.1903, −0.0604) **
	*CC + CT*	462 (75.24)	reference	reference
	*TT*	152 (24.76)	−0.1595 (−0.2533, −0.0656) **	−0.0914 (−0.1451, −0.0377) **
MTHFR *A1298C*	*CC*	27 (4.40)	reference	reference
	*CA*	177 (28.83)	0.0484 (−0.1512, 0.2480)	−0.0114 (−0.1237, 0.1009)
	*AA*	410 (66.78)	−0.0154 (−0.1116, 0.0809)	−0.1264 (−0.2347, −0.0181) *
	*CC + CA*	204 (33.22)	reference	reference
	*AA*	410 (66.78)	−0.0728 (−0.1560, 0.0105) +	−0.1165 (−0.1633, −0.0670) **
MTRR *A66G*	*GG*	39 (6.35)	reference	reference
	*GA*	234 (38.11)	−0.0497 (−0.2164, 0.1169)	−0.0211 (−0.1162, 0.0740)
	*AA*	341 (55.54)	−0.0216 (−0.1033, 0.0600)	−0.0572 (−0.1503, 0.0360)
	*GG*	39 (6.35)	reference	reference
	*GA + AA*	575 (93.65)	−0.0460 (−0.2055, 0.1136)	−0.0422 (−0.1334, 0.0490)
MTR *A2756G*	*AA*	501 (81.60)	reference	reference
	*AG*	103 (16.78)	−0.1329 (−0.2360, −0.0298) *	0.0264 (−0.0329, 0.0857)
	*GG*	10 (1.63)	−0.2162 (−0.5201, 0.0877)	−0.0440 (−0.2188, 0.1308)
	*AA*	501 (81.60)	reference	reference
	*AG + GG*	113 (18.40)	−0.1402 (−0.2395, −0.0410) *	0.0202 (−0.0369, 0.0773)

^a^. Adjusted for age, gender, BMI, birth weight, and province; ^b^. A linear regression model was employed to assess the relationship; +: 0.05 < *p* < 0.1; *: 0.001 < *p* < 0.05; **: *p* ≤ 0.001.

**Table 4:** Associations of joint gene polymorphisms with serum folate level/cognitive scores.

Variables		n (%)	Folate-Adjusted β (95%CI) ^a,b^	Cognition-Adjusted β (95%CI) ^a,b^
MTHFR *C677T*	MTHFR *A1298C*			
*CC + CT*	*CC + CA*	200 (32.57)	reference	reference
*CC + CT*	*AA*	262 (42.67)	−0.0177 (−0.1070, 0.0717)	−0.1001 (−0.1506, −0.0495) **
*TT*	*CC + CA*	4 (0.65)	0.1649 (−0.3158, 0.6456)	−0.0325 (−0.3044, 0.2393)
*TT*	*AA*	148 (24.10)	−0.1788 (−0.2872, −0.0704) *	−0.1538 (−0.2151, −0.0924) **
MTHFR *C677T*	MTRR *A66G*			
*CC + CT*	*GG*	30 (4.89)	reference	reference
*CC + CT*	*GA + AA*	432 (70.36)	−0.0739 (−0.2542, 0.10664)	−0.0202 (−0.1232, 0.0828)
*TT*	*GG*	9 (1.47)	−0.2754 (−0.6398, 0.0890)	−0.0026 (−0.2109, 0.2057)
*TT*	*GA + AA*	143 (23.29)	−0.2264 (−0.4216, −0.0313) *	−0.1169 (−0.2284, −0.0053) *
MTHFR *C677T*	MTR *A2756G*			
*CC + CT*	*AA*	380 (61.89)	reference	reference
*CC + CT*	*AG + GG*	82 (13.36)	−0.1380 (−0.2531, −0.0229) *	0.0523 (−0.0137, 0.1184)
*TT*	*AA*	121 (19.71)	−0.1576 (−0.2613, −0.0540) *	−0.0708 (−0.1303, −0.0113) *
*TT*	*AG + GG*	31 (5.05)	−0.2812 (−0.4595, −0.1028) *	−0.1253 (−0.2276, −0.0230) *
MTHFR *A1298C*	MTRR *A66G*			
*CC + CA*	*GG*	9 (1.47)	reference	reference
*CC + CA*	*GA + AA*	195 (31.76)	−0.1806 (−0.5072, 0.1460)	0.0290 (−0.1545, 0.2124)
*AA*	*GG*	30 (4.89)	−0.2312 (−0.5942, 0.1318)	−0.0138 (−0.2177, 0.1901)
*AA*	*GA + AA*	380 (61.89)	−0.2466 (−0.5701, 0.0770)	−0.0948 (0.2765, 0.0869)
MTHFR *A1298C*	MTR *A2756G*			
*CC + CA*	*AA*	163 (26.55)	reference	reference
*CC + CA*	*AG + GG*	41 (6.68)	−0.1548 (−0.3211, 0.0115) +	0.0707 (−0.233, 0.1646)
*AA*	*AA*	338 (55.05)	−0.0802 (−0.1721, 0.0118) +	−0.0996 (−0.1516, −0.0477) **
*AA*	*AG + GG*	72 (11.73)	−0.2172 (−0.3528, −0.0815) *	−0.1144 (−0.1911, −0.0378) *
MTRR *A66G*	MTR *A2756G*			
*GG*	*AA*	34 (5.54)	reference	reference
*GG*	*AG + GG*	5 (0.81)	0.2607 (−0.1959, 0.7172)	−0.0129 (−0.2760, 0.2503)
*GA + AA*	*AA*	467 (76.06)	0.0195 (−0.1508, 0.1898)	−0.0485 (−0.1466, 0.0497)
*GA + AA*	*AG + GG*	108 (17.59)	−0.1406 (−0.3280, 0.0468)	−0.0253 (−0.1333, 0.0827)

^a^. Adjusted for age, gender, BMI, birth weight, and province; ^b^. A linear regression model was employed to assess the relationship; +: 0.05 < *p* < 0.1; *: 0.001< *p* < 0.05; **: *p* ≤ 0.001.

**Table 5:** Relationship between the number of risk genotypes and serum folate/cognitive scores.

Number of Risk Genotypes ^a^	Total (n = 614)		Serum Folate Level	
	Folate-β (95%CI) ^b,c^	Cognition-β (95%CI) ^b,c^	Low and Medium (*n* = 512)	High (*n* = 102)
			Cognition-β (95%CI) ^b,c^	Cognition-β (95%CI) ^b,c^
0 (*n* = 154)	reference	reference	reference	reference
1 (*n* = 247)	−0.0373 (−0.1349, 0.0604)	−0.0620 (−0.1178, −0.0061) *	−0.0567 (−0.1178, 0.0044) +	−0.0966 (−0.2405, 0.0474)
2 (*n* = 172)	−0.1504 (−0.2583, 0.0425) *	−0.1049 (−0.1666, −0.0431) **	−0.0899 (−0.1557, −0.0241) *	−0.2231 (−0.4119, −0.0343) *
3 (*n* = 41)	−0.2617 (−0.4312, 0.0921) *	−0.1351 (−0.2321, −0.0381) *	−0.1019 (−0.2041, 0.0003) +	−0.3644 (−0.6750, −0.0539) *

^a^. Risk genotypes were defined as MTHFR *677TT*, MTHFR *1298AA*, MTRR *66 GA + AA*, and MTR *2756AG + GG*; ^b^. Adjusted for age, gender, BMI, birth weight, and province; ^c^. A linear regression model was employed to assess the relationship. +: 0.05 < *p* < 0.1; *: 0.001 < *p* < 0.05; **: *p* ≤ 0.001.

## Data Availability

The datasets generated and analyzed during the current study are not publicly available due to participant privacy protection, but are available from the corresponding author on reasonable request.

## Supplementary Materials

The following supporting information can be downloaded at: https://www.mdpi.com/article/10.3390/nu17162666/s1, Guidance S1: Guidance Manual for Primary Testers of Basic Cognitive Skills.

## Abbreviations

The following abbreviations are used in this manuscript:

DHF	dihydrofolate
THF	tetrahydrofolate
5,10-CH_2_-THF	5,10-methylenetetrahydrofolate
5-MTHF	5-methyltetrahydrofolate
MTHFR	5,10-methylenetetrahydrofolate reductase
MTR	5-methyltetrahydrofolate transmethylase
MTRR	5-methyltetrahydrofolate transmethylase reductase
SNP	single-nucleotide polymorphisms
LHEAITNP	the Long-term Health Effects Assessment Project of Infants and Toddlers Nutritional Pack
KASP	Kramer’s allele-specific PCR
K-ABC	Kaufman assessment battery for children
GLM	generalized linear model
