# Supplementary material for: Synergistic Effects of MTHFR, MTRR, and MTR Gene Variants on Serum Folate Levels and Cognitive Function in Chinese Preschoolers: A Cross-Sectional Study
Source: Nutrients. 2025 Aug 18;17(16):2666. doi: 10.3390/nu17162666 (PMC12389356; doi:10.3390/nu17162666)

Guidance Manual for Primary Testers of Basic Cognitive Skills

Printed July 2023

Part I: Graphical reasoning（6-minute time limit）

Children are shown incomplete patterns and are asked to choose one of five alternatives that fills in the missing part of the pattern, which is then filled in on the answer key by the main test taker. Specific instructions are given below:

You will see some incomplete patterns like this, and there are five alternative answers below, from which you need to choose the one that fills in the "? " part of the pattern. Wait for children to answer ...

Yes, the answer is 1. All the butterflies here are blue, so you should put this graphic (option 1) here (point to the “?”). The answer is 1, all the butterflies here are blue.

Here's another example question. Which one (of the five alternative choices) should go here (point to the “?” position). Wait for the child to answer ...

Yes, the answer is 5. These two bulbs are yellow (point to the two yellow bulbs) and this one is green (point to the green bulb), so you should put this green bulb (point to option 5) here (point to the “?”). The green bulb (point to option 5) should be placed here (point to the “?”).

Look at another example question. Which one (sweep your finger over the five alternatives) should go here (point to the “?” position) and wait for the child to answer ... " position) and wait for the child to answer ...

Yes, the answer is 4. All the shapes are blue with this slash (point to the incomplete pattern above), so you should put this blue one (point to option 4) with the same slash here (point to the “?”).

This is followed by a formal test in which children are asked to answer the questions one at a time and a timer is started. If they are not quite sure how to answer, the master tester tries to guide them simply by saying, “Which one (sweep your finger across the five answer choices in the bottom row) goes here (point your finger to the ”?" box in the matrix)? (point your finger to the “?” box in the matrix). When children have an understanding of how to answer the questions, the primary tester can shorten or omit the directions and just fill out the answer key.

Abort Rule: The quiz will be aborted when the 6 minutes are up or when there are three consecutive 0 scores.

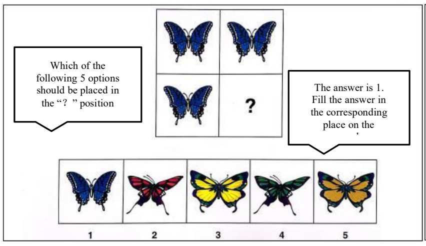

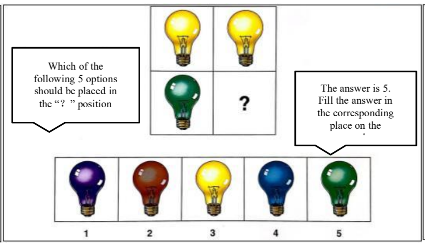

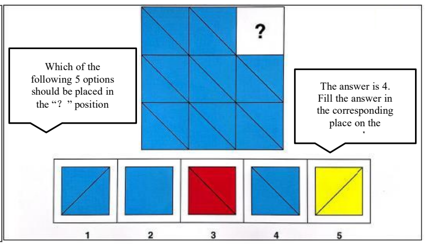

Part II: Reciting Numbers in Reverse Order

A series of numbers is read to the child at a rate of one number per second and the child is asked to say the series of numbers read by the test subject in reverse order. The instructions are as follows:

The tester said, "Now, I'm going to read some numbers, so please pay attention, and when I'm done, you're going to memorize them in reverse order. For example, if I say 8 - 2, what should you say?" If the child answers correctly (2 - 8); the tester should say, "Yes, that's right." If the answer is incorrect, the tester should say, "No, I said 8 - 2, you have to say it backwards, it should be 2 - 8," and move on to the next example question.

The master test says, "Let's try it again, and remember, you have to memorize it in reverse order. Listen carefully: 5 - 6." If the child gets the answer right (6 - 5); the master should say, "Yes, that's it." If the answer is incorrect, the test subject should say, "No, I said 5 - 6, but you have to say it backwards, it should be 6 - 5." The test subject then proceeds to the formal administration of the test, where the test subject does not give feedback on the child's answer. Regardless of whether it is right or wrong, the test subject may say, "Okay, next question."

Abort Rule: A score of 0 on both attempts of the same breadth will abort the quiz.

Notes:

1) Read at a rate of one number per second, slightly increasing and dragging out the tone of your voice when you reach the last number. Pause for a few seconds after each series of numbers to allow the child to respond.

2) Each question was given two attempts. Whether or not the child passes the first attempt, a second attempt is administered.

3) No question can be repeated. If the child asks for another reading, the master test taker should say, "Guess as much as you can."

Part II: Reciting Numbers in Reverse Order (score)

Serial number	Reciting Numbers in Reverse Order	Score	Total score
Example	8 – 2		
	5 – 6		
1	2 – 1	0 1	0 1 2
	1 – 3	0 1	
2	3 – 5	0 1	0 1 2
	6 – 4	0 1	
3	5 – 7 – 4	0 1	0 1 2
	2 – 5 – 9	0 1	
4	7 – 2 – 9 – 6	0 1	0 1 2
	8 – 4 – 9 – 3	0 1	
5	4 – 1 – 3 – 5 – 7	0 1	0 1 2
	9 – 7 – 8 – 5 – 2	0 1	
6	1 – 6 – 5 – 2 – 9 – 8	0 1	0 1 2
	3 – 6 – 7 – 1 – 9 – 4	0 1	
7	8 – 5 – 9 – 2 – 3 – 4 – 6	0 1	0 1 2
	4 – 5 – 7 – 9 – 2 – 8 – 1	0 1	
8	6 – 9 –1 – 7 – 3 – 2 – 5 – 8	0 1	0 1 2
	3 – 1 – 7 – 9 – 5 – 4 – 8 – 2	0 1	
9	2 – 7 – 6 – 4 – 1 – 3 – 9 – 5 – 6	0 1	0 1 2
	5 – 2 – 3 – 7 – 6 – 8 – 4 – 9 – 3	0 1	

Part I：Decoding（120-second time limit）

Place the “Part 3 Decoding” answer key in front of the child. Show the child the Figure-Symbol Correspondence Table and ask the child to draw the symbols inside the figures according to the correspondences in the table within the given time limit. Specific instructions are given below:

Here are some graphs (finger the graph-symbol correspondence table), each with a different symbol drawn inside.

Look, there's a circle here (point to the first example), and the circle above it (point to the circle in the corresponding table) has two horizontal lines in it, so I'm going to draw two horizontal lines in this one as well (the tester starts drawing).

Look again, there is a pentagram here (pointing to the second example), and the pentagram above it (pointing to the pentagram in the corresponding table) has a vertical line inside it, so I'm going to draw a vertical line inside this pentagram as well (the tester starts drawing).

(Give the child a 2B pencil without an eraser tip.) Now, try to do the questions here (point to the remaining exercises), stopping at this line (point to the vertical line after the example). Then, have children complete the remaining parts of the example independently. If correct, the primary tester can encourage “right/good”; if there are mistakes, the primary tester can correct them immediately.。

(After children have completed the exercise) When I say start, you begin to draw from here (point to the official first question). Remind again, "Follow the order and don't skip any questions. Draw as fast as you can, but get it right." After the test subject has prepared a stopwatch, have the child begin. If the child makes a mistake, the child is told, “It's okay, just move on as fast as you can.” The child is asked to stop answering after 120 seconds, and a score is given for the number of correct completions in 120 seconds (59 total). If the child finishes early, the tester records the completion time and adds points according to the table below.

Table. Score of Decoding Speed（Out of 65 points）

Completion time（second）	Bonus points	Scores（Plus bonus points）
116～120	0	59
111～115	1	60
106～110	2	61
101～105	3	62
96～100	4	63
86～95	5	64
≤85	6	65

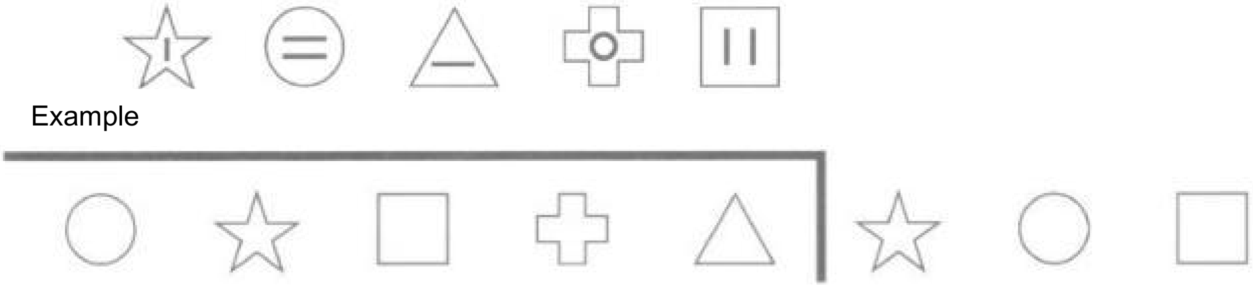

Children's Basic Cognitive Assessment Answer Key

Part I: Graphical reasoning (score)

Example 1	Example 2	Example 3	1	2	3	4	5	6	7
1	5	4	3	1	2	4	3	5	1

8	9	10	11	12	13	14	15	16	17
2	4	4	3	4	1	1	2	4	1

Part II: Reciting Numbers in Reverse Order (score)

Serial number	Reciting Numbers in Reverse Order	Score	Total score
Example	8 – 2		
	5 – 6		
1	2 – 1	0 1	0 1 2
	1 – 3	0 1	
2	3 – 5	0 1	0 1 2
	6 – 4	0 1	
3	5 – 7 – 4	0 1	0 1 2
	2 – 5 – 9	0 1	
4	7 – 2 – 9 – 6	0 1	0 1 2
	8 – 4 – 9 – 3	0 1	
5	4 – 1 – 3 – 5 – 7	0 1	0 1 2
	9 – 7 – 8 – 5 – 2	0 1	
6	1 – 6 – 5 – 2 – 9 – 8	0 1	0 1 2
	3 – 6 – 7 – 1 – 9 – 4	0 1	
7	8 – 5 – 9 – 2 – 3 – 4 – 6	0 1	0 1 2
	4 – 5 – 7 – 9 – 2 – 8 – 1	0 1	
8	6 – 9 –1 – 7 – 3 – 2 – 5 – 8	0 1	0 1 2
	3 – 1 – 7 – 9 – 5 – 4 – 8 – 2	0 1	
9	2 – 7 – 6 – 4 – 1 – 3 – 9 – 5 – 6	0 1	0 1 2
	5 – 2 – 3 – 7 – 6 – 8 – 4 – 9 – 3	0 1	

Part III： **Decoding (Completion time + Number of correct completions + Speed bonus = Final score)**

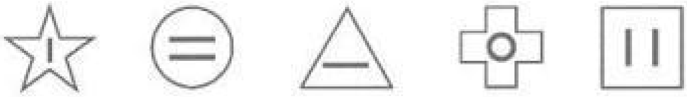

Example

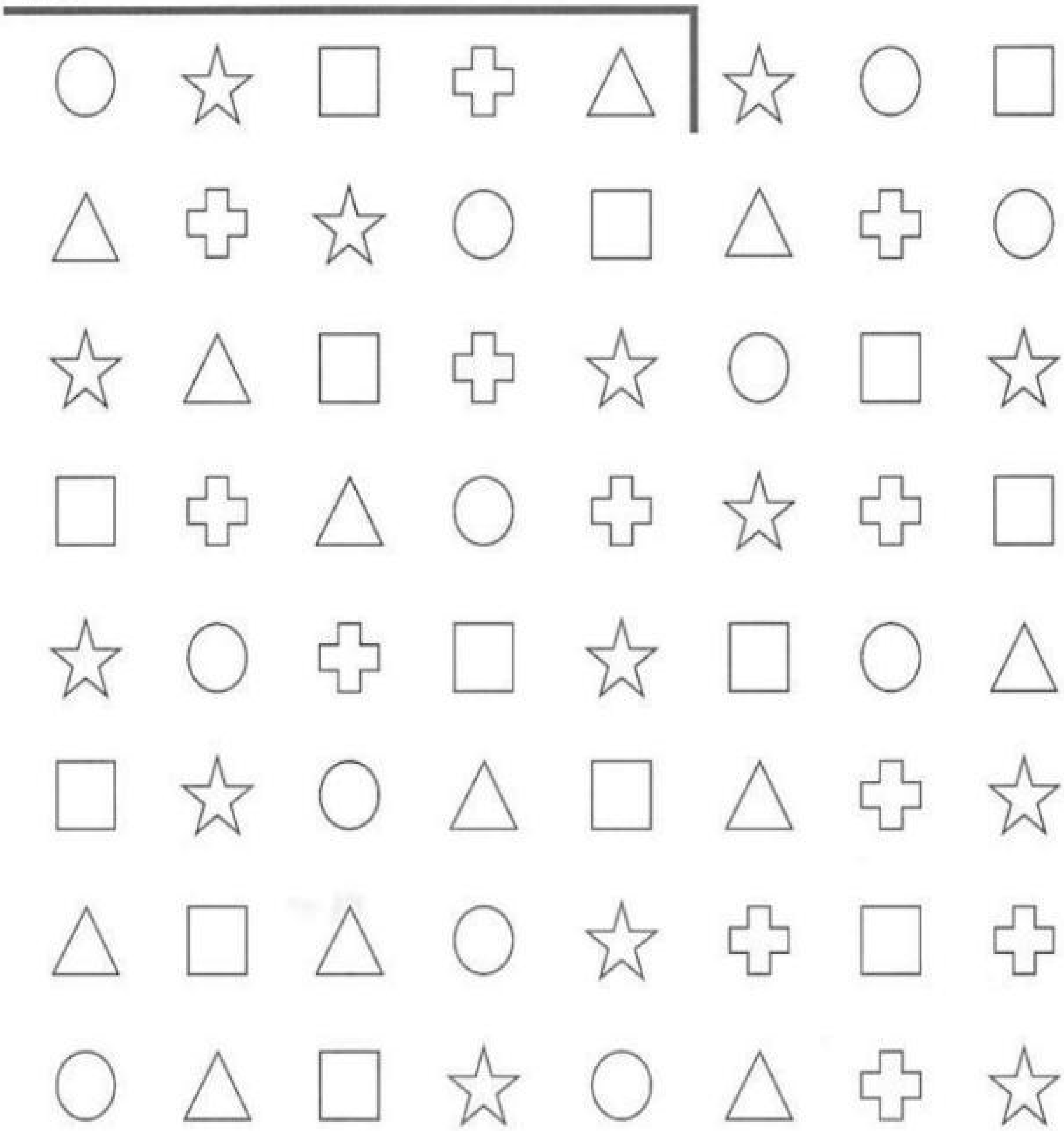

Supplement: Supplementary file 1 [file nutrients-17-02666-s001.zip › nutrients-3769245-supplementary.pdf]
